# Medical-Grade Physical Activity Monitoring for Measuring Step Count and Moderate-to-Vigorous Physical Activity: Validity and Reliability Study

**DOI:** 10.2196/10706

**Published:** 2018-09-05

**Authors:** Myles William O'Brien, William Robert Wojcik, Jonathon Richard Fowles

**Affiliations:** ^1^ Centre of Lifestyle Studies, School of Kinesiology Acadia University Wolfville, NS Canada; ^2^ Division of Kinesiology Dalhousie University Halifax, NS Canada

**Keywords:** pedometer, accelerometry, exercise prescription, validity, reliability

## Abstract

**Background:**

The use of physical activity (PA) monitors is commonly associated with an increase in habitual PA level in healthy and clinical populations. The PiezoRx is a medical-grade PA monitor that uses adjustable step rate thresholds to estimate moderate-to-vigorous physical activity (MVPA) and is a valid indicator of free-living PA in adults. Laboratory validation of step count derived MVPA in adults is needed to justify the use of these monitors in clinical practice to track individuals’ progress toward meeting PA guidelines that are based on MVPA, not steps.

**Objective:**

The objective of our study was to assess the validity and interinstrument reliability of the PiezoRx to derive step count and MVPA in a laboratory setting compared with criterion measures and other frequently used PA monitors in a diverse sample of adults.

**Methods:**

The adult participants (n=43; 39.4 years, SD 15.2) wore an Omron HJ-320 pedometer, an ActiGraph GT3X accelerometer, and four PiezoRx monitors during a progressive treadmill protocol conducted for 6 minutes at speeds of 2.4, 3.2, 4.0, 5.6, 6.4, and 7.2 km/hour, respectively. The four PiezoRx monitors were set at different MVPA step rate thresholds (MPA in steps/minute/VPA in steps/minute) 100/120, 110/130, height adjusted, and height+fitness adjusted.

**Results:**

The PiezoRx was more correlated (intraclass correlation, ICC=.97; *P*<.001) to manual step counting than the ActiGraph (ICC=.72; *P*<.001) and Omron (ICC=.62; *P*<.001). The PiezoRxs absolute percent error in measuring steps was 2.2% (ActiGraph=15.9%; Omron=15.0%). Compared with indirect calorimetry, the height-adjusted PiezoRx and ActiGraph were accurate measures of the time spent in MVPA (both ICC=.76; *P*<.001).

**Conclusions:**

The PiezoRx PA monitor appears to be a valid and reliable measure of step count and MVPA in this diverse sample of adults. The device’s ability to measure MVPA may be improved when anthropometric differences are considered, performing at par or better than a research grade accelerometer.

## Introduction

Regular physical activity (PA) and exercise are associated with a reduced risk of developing cardiovascular diseases, cancer, and diabetes mellitus [1]. However, most Canadians do not engage in a sufficient level of daily activity with only 15% meeting the PA guidelines of 150 minutes of moderate-to-vigorous aerobic physical activity (MVPA) per week and 35% achieving 10,000 steps per day [2]. The use of PA monitors is one way of assisting individuals in increasing their PA; meta-analyses of randomized controlled trials have shown that the use of PA monitors increased the daily activity of the healthy and diseased populations by 1800-2500 steps per day [3,4]. Moreover, step count prescriptions provided by physicians increased the daily step count by 20% and improved the glycemic control of patients with type 2 diabetes and hypertension after 1 year compared with the control group [5]. Several primary care providers identify that the lack of tangible aids (ie, pedometers and resources to support PA) is a major barrier in prescribing exercise [6]. Health care providers may not use PA monitors as a measurement tool because of their accuracy and reliability, particularly owing to the different capabilities of popular commercial grade PA monitors in measuring step count and MVPA [7]. Considering the accumulating evidence on the benefits of PA and the clinical outcomes of PA monitoring, the validity and reliability of such patient-focused PA monitors must be evaluated. A greater confidence in the measurement of accuracy may lead to an increase in the use of PA monitors among health care professionals.

Recently, a new piezoelectric PA monitor (PiezoRx, StepsCount Inc, Deep River, ON, Canada) has been approved as a class 1 medical device by Health Canada, and this device may be prescribed by health care providers to their patients for monitoring PA. Moreover, the device uses adjustable step rate thresholds to determine the time spent performing MVPA. Factory settings of 100 steps per minute (spm) and 120 spm were identified to correspond to moderate physical activity (MPA; 3 metabolic equivalents, METs) and vigorous physical activity (VPA; 6 METs), respectively, based on previous indications for these thresholds from the literature [8-10]. However, the MVPA step rate thresholds differ when considering individual anthropometric (ie, height) and aerobic fitness differences [10-12]. Whether adjusting the MVPA step rate thresholds according to factors known to influence step rate bioenergetics improves the validity of the PiezoRx in measuring MVPA is not known. A recent pilot study has shown that the height-adjusted PiezoRx can accurately measure step count and MVPA in a diverse sample of adults in free-living conditions compared with accelerometry [13]. However, the use of the ActiGraph as a criterion measure of MVPA may produce some errors compared with direct counting of steps and indirect calorimetry. In addition, only a single-adjusted PiezoRx was used, and whether other MVPA thresholds are more accurate is unclear. Therefore, the accuracy of multiple PiezoRx devices set at individualized MVPA thresholds must be compared with that of indirect calorimetry in a controlled laboratory setting.

Previous laboratory validation studies have used the older versions of the device (SC-StepMX and SC-StepRx), and results have shown that the device was better than research grade PA monitors in measuring the step count of healthy children and young adults [14], adults [15], and older adults [16]. When the MVPA step rate thresholds (MPA/VPA) were increased to 110/130, the PiezoRx and a research grade accelerometer had similar measurements for MVPA compared with indirect calorimetry in children and young adults [14], although no study to date has compared the accuracy of the devices and indirect calorimetry in measuring MVPA in adult populations.

Considering the potential of a medical-grade PA monitor to assist health care providers in prescribing exercise and monitoring the PA of their patients, the accuracy of such devices in measuring step count and MVPA must be evaluated. Therefore, this study aimed to assess the validity and interinstrument reliability of these devices in measuring steps and MVPA during a progressive treadmill walking protocol in a diverse sample of adults.

## Methods

### Participants

A sample of 43 adults (25 women) aged between 20 and 64 years (mean age 39.4 years, SD 15.2) volunteered to participate in this study. The average body mass index (BMI) and aerobic fitness of the participants were 27.9 kg/m^2^ (SD 6.1) and 41.2 mL/kg/min (SD 10.2), respectively. The majority (n=24) of participants answered “yes” to at least one question in the Physical Activity Readiness Questionnaire Plus (PAR-Q+), and they were cleared for MVPA [17]. Moreover, they completed the Canadian Society for Exercise Physiology-Physical Activity and Sedentary Behavior Questionnaire (CSEP-PASB-Q), which is a valid and reliable measurement tool for weekly MVPA [18]. The participants self-reported their aerobic fitness levels based on PASB-Q as poor (n=1), fair (n=6), good (n=12), very good (n=13), and excellent (n=11). All participants were recruited via a community-wide email and by word of mouth, and a written informed consent was obtained from the participants. The study was conducted in Wolfville, Nova Scotia, from May 2015 to September 2015, and it was approved by the research ethics board of Acadia University (REB# 15-20).

### Experimental Design

After the prescreening procedures, anthropometric (ie, height and weight) measurements and aerobic fitness were evaluated by a Canadian Society for Exercise Physiology-Certified Personal Trainer according to published guidelines [19,20]. Aerobic fitness was predicted using the submaximal Ebbeling protocol [21], as described in more detail below. After the submaximal aerobic test, a resting period of 20-30 minutes was allotted to ensure that the participants returned to a rested state; then, four PiezoRxs, one Omron HJ-320, and one ActiGraph GT3X accelerometer were placed around the waist of the participants according to manufactures’ recommendations. Thereafter, the participants were asked to complete the multistage treadmill walking protocol to assess and compare the validity of the ActiGraph, Omron, and PiezoRx with finite step rate thresholds (ie, 100/120 and 110/130) and individualized step rate thresholds (ie, height adjusted and height+fitness adjusted) with that of the criterion measures of manual step counting and indirect calorimetry.

### Aerobic Fitness

Aerobic fitness was estimated using the Ebbeling walking treadmill protocol [21]. The Ebbeling consists of two 4-min walking stages. The first stage is designed to reach a speed that elicits 60% of the participants’ maximum estimated heart rate (ie, 220–age). The second stage included the increase in the incline by 5% and maintenance of the previously established speed. Treadmill speed and steady-state heart rate were used to estimate VO_2 max_ using a prediction equation [20]. A submaximal test was chosen over a maximal test for safety reasons and a minimal influence after the walking assessment because it was most practical to complete the fitness testing and step assessment in a single session.

### Physical Activity Monitors

Each participant used four PiezoRx pedometers (StepsCount, ON, Canada), one ActiGraph GT3X accelerometer (ActiGraph, FLA, the USA), and one Omron HJ-320 pedometer (Omron Healthcare, Kyoto, Japan) that were attached on an adjustable leather belt around their waist. All devices were worn in accordance with the manufactures’ recommendations. In particular, two PiezoRx and ActiGraph were fitted in line with the left thigh, whereas the other two PiezoRx and Omron were fitted in line with the right thigh. The ActiGraph accelerometer was sampled at 30 Hz and was initialized to collect data using 15-second epochs. The ActiGraph cut point for MVPA was >1952 counts [22]. The PiezoRx thresholds for monitoring MVPA were set as follows: 100/120, 110/130, adjusted for height, and adjusted for height+fitness (Table 1). The PiezoRxs thresholds that were set at 100/120 and 110/130 were based on the current literature that consistently showed that 100-110 spm is equal to ~3 METs (ie, 10.5 mL/min/kg) and that 120-130 spm is equal to ~6 METs (ie, 21 mL/min/kg) in adults [8-11,23,24].

Adjustments recommended for height and fitness were based on previous literature and unpublished results in our laboratory indicating height and fitness related changes in step rate bioenergetics [10-12]. For these adjustments, baseline step thresholds were chosen based on the available literature indicating that 100 and 130 spm are heuristic cadence-intensity thresholds for MPA and VPA, respectively [24].

An adjustment of 5 spm is appropriate for every 10 cm increase (ie, 5 spm lower) or 10 cm decrease in height (ie, 5 spm higher) based on the premise that shorter individuals must take more steps to cover the same distance and therefore similar external work. In addition, considering the known impact of cardiorespiratory fitness on step rate bioenergetics [12], aerobic fitness was proportionally associated with 10 spm adjustments to step rate thresholds. For example, a VO_2 max_ of “Excellent” [19] resulted in an increase in MPA and VPA step rate thresholds by 10 spm (eg, 110/140; see Table 1).

The distributions of MVPA step rate thresholds for the height-adjusted PiezoRx were as follows: 90/120 (n=1), 95/125 (n=11), 100/130 (n=10), 105/135 (n=17), and 110/140 (n=4). The distributions of MVPA step rate thresholds for the height+fitness-adjusted PiezoRx were as follows: 90/120 (n=2), 95/125 (n=13), 100/130 (n=6), 105/135 (n=13), 110/140 (n=7), and 115/145 (n=2).

### Exercise Protocol

Once the participants were equipped with the activity monitors, they were fitted with a headpiece, two-way nonrebreathing valve (Hans Rudolph, Inc, KS, the USA), noseclip, and mouthpiece. The metabolic cart (TrueOne 2400; Parvo Medics, UT, USA) was calibrated using nitrogen and two primary standard gas mixtures to an error rate of 0.01%. The pneumotachometer was calibrated using a 3-L syringe that delivered fixed volumes at different flow rates. Volume calibration was <0.1 L. They proceeded to complete a 6-stage treadmill protocol at predetermined speeds of 2.4, 3.2, 4.0, 5.6, 6.4, and 7.2 km/h at 0% grade, which correlated to a metabolic intensity average of 2.6 (SD 0.4), 2.8 (SD 0.4), 3.1 (SD 0.4), 4.3 (SD 0.4), 5.3 (SD 0.4), and 6.8 (SD 0.7) METs for each stage, respectively. Each stage consisted of 6 min of walking to achieve a metabolic steady state, followed by 4 min of rest. The steady-state VO_2_ was used as stage VO_2_ in the analysis to limit the variability introduced by oxygen kinetics during the onset of exercise in each stage. Steps were manually counted by two instructors for 2-3 and 4-5 min during each stage to determine the step count criterion. A video camera was used to film the feet of the participant in case the testers recorded >1 step difference during a stage. The 6-stage protocol corresponded to stepping cadences of 86 (SD 8), 97 (SD 7), 106 (SD 7), 120 (SD 7), 128 (SD 7), and 139 (SD 8) spm for each stage, respectively. Data from the PiezoRx and Omron monitors were extracted during the 4-min rest phase with the participant straddling the treadmill, and the devices were reset prior to the next stage. Data from the ActiGraph were extracted at the end of the exercise. At the end of each stage, the participants immediately straddled the treadmill. Immediately prior to the start of the next stage, the treadmill belt speed was increased to the necessary speed to avoid differences in step count and MVPA that may occur during treadmill acceleration or deceleration. The test was terminated because of volitional fatigue if the participant reached 85% of his or her estimated maximum heart rate (220 – age) or if they finished all 6 stages of the protocol. An appropriate cool down was administered by the instructor while monitoring their heart rate recovery.

### Data and Statistical Analyses

Data were analyzed using the Statistical Package for Social Sciences software version 23.0 (IBM, NY, USA). A *P* value <.05 was considered statistically significant. Manually counted steps and MVPA obtained via indirect calorimetry were assessed for normality using the Shapiro-Wilk test. Manually counted steps were normally distributed (*P*>.05). However, MVPA obtained via indirect calorimetry were not normal owing to the number of walking stages composed of either 0 or 360 sec of MVPA. Only the PiezoRx set at factory settings was used for the validity of the step count analysis. The steps counted within 2-3 and 4-5 min for each stage were averaged and multiplied by a factor of 6 to determine the number of steps for each 6-min stage. In relation to this, the steps counted within 2-3 and 4-5 min were always within 2 step/min.

A two-way mixed-model intraclass correlation coefficients (ICC) with measures of consistency were utilized to measure the strength and consistency of each device’s capability to measure steps with those of the criterion measure of manual step counting. The Lin’s concordance coefficients (LCC) and Bland-Altman plots (BAPs) were utilized to assess the agreement between monitors and the criterion measures [25,26].

**Table 1:** Algorithm used to determine the PiezoRx height- and fitness-adjusted moderate-to-vigorous physical activity step rate thresholds.

Height, cm (ft in)	Height adjusted (MPA/VPA^a^ thresholds)	Fitness classification^b^ (MPA/VPA thresholds)		
		Poor/fair	Good/very good	Excellent
140 (4’8”)	115/145	105/135	115/145	125/155
150 (5’0”)	110/140	100/130	110/140	120/150
160 (5’3”)	105/135	95/125	105/135	115/145
170 (5’7”)	100/130	90/120	100/130	110/140
180 (5’11”)	95/125	85/115	95/125	105/135
190 (6’3”)	90/120	80/110	90/120	100/130
200 (6’6”)	85/115	75/105	85/115	95/125

^a^MPA/VPA: moderate physical activity/vigorous physical activity.

^b^The ratings for aerobic fitness were based on normative values from the Canadian Society for Exercise Physiology (CSEP) physical activity and training for health.

BAPs were also generated based on the steps measured using the PiezoRx, ActiGraph, and Omron to determine the mean bias (ie, overpredicted or underpredicted values) and the limits of agreement (LoA; SD 95% of mean bias). A 4×6 (device×stage) repeated measure analysis of variance (RM-ANOVA) was used to assess the differences between the PiezoRx, Omron, and ActiGraph steps as well as manual step counting across treadmill stages. The absolute percent error (APE) of the PiezoRx, ActiGraph, and Omron was calculated using the equation: (|manually counted − device measured|)/(manually counted) × 100%. The reliability of the PiezoRx step count function was assessed using ICC and RM-ANOVA (device×stage) using all four devices. Bonferroni post hoc testing was carried out for all statistically significant RM-ANOVAs.

For each device and the metabolic cart, the amount of time spent in performing MVPA for each walking stage was summed up for full 6 min for analysis. The absolute MVPA difference per stage (metabolic cart-device measured) and Spearman correlation coefficients (SCCs) were used to assess and compare the validity of the four PiezoRx devices and the ActiGraph accelerometer in measuring MVPA with that of indirect calorimetry. BAP was used to assess the agreement between the height-adjusted PiezoRx and both indirect calorimetry and the ActiGraph in obtaining MVPA measurement. The difference in the absolute MVPA per stage (sec/stage) was calculated over an absolute percent change owing to the dichotomous nature of exercise stages (either the absence of time spent in MVPA or comprised entirely of time spent in MVPA). As such, the sensitivity and specificity rates of each device were calculated to identify if the stage has moderate-to-vigorous intensity compared with the metabolic cart. A stage was considered to have a moderate-to-vigorous intensity if at least 80% of the stage (ie, 288 sec) was greater than 3 METs (ie, 10.5 mL/kg/min).

The reliability of MVPA measurement obtained using the PiezoRx was assessed using the absolute MVPA difference per stage (|metabolic cart − device measured|) and SCCs for each stage where the height-adjusted and height+fitness-adjusted MVPA thresholds were equal (see Table 1; n=25).

## Results

### Validity and Reliability of Step Count Measurement

**Figure 1:**
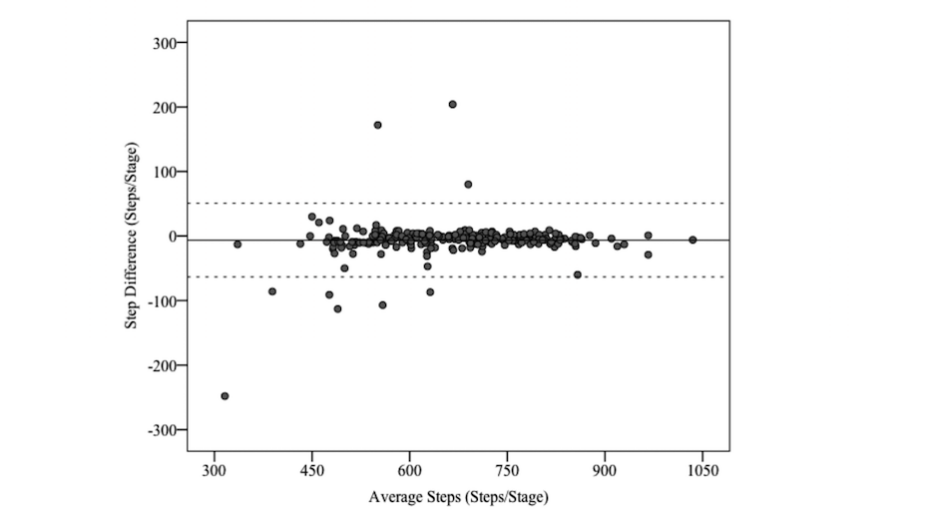
Bland-Altman plot analysis for manual step counting and the PiezoRx-determined step count.

Compared with manual step counting, the PiezoRx had a higher intraclass correlation (ICC=.97; *P*<.001) and lower percent error (2.2%, SD 5.4), than both the ActiGraph (ICC=.72, *P*<.001; APE=15.9%, SD 26.8) and the Omron (ICC=.62, *P*<.001; APE=15.0%, SD 29.0), as shown in Table 2, Table 3, and Table 4. As shown in Figure 1, Bland-Altman analysis revealed a fixed bias of −6.4 steps (*P*=.001) with LoA of −63 and 51 steps for the PiezoRx and manually counted steps. Step count differences were observed between manual step counting and both the ActiGraph and Omron during the first stage (2.4 km/h; both *P*<.001) and second stage (3.2 km/h; ActiGraph: *P*<.001; Omron: *P*=.04) but only the Omron during the fourth stage (5.6 km/h; Omron: *P*=.046).

ICC using all four devices showed an ICC interinstrument reliability of 0.88 (*P*<.001), as shown in Table 5. All four PiezoRx devices were included in the reliability of step count analysis. Cases in which the height- and height/fitness-adjusted step rate thresholds were identical were used for the MVPA reliability analysis. The correlation was stronger (ICC>.90) during the fastest walking stages (6.4-7.2 km/h) and was weakest (ICC<.50) during the middle speed stages (4.0-5.6 km/h). RM-ANOVA revealed no significant (*P*>.05) differences between all four PiezoRx devices for measuring step count.

### Validity and Reliability of Moderate-to-Vigorous Physical Activity

The devices with the highest Spearman correlations in measuring MVPA across all stages were the height-adjusted PiezoRx (SCC=.80; *P*<.001) and the ActiGraph (SCC=.80; *P*<.001), which also had similar absolute time differences per 6-minute walking stage (height-adjusted PiezoRx: 53±93 sec/stage; ActiGraph: 53±103 sec/stage). MVPA SCC of the PiezoRx (100/120), PiezoRx (110/130), and PiezoRx (height+fitness adjusted) were 0.74 (*P*<.001), 0.76 (*P*<.001), and 0.77 (*P*<.001), respectively.

**Table 2:** Two-way mixed-model intraclass correlation with measure of consistency.

Walking speed (km/h)	n	PiezoRx			Actigraph			Omron	
		Intraclass correlation (95% CI)^a^	*P* value	Intraclass correlation (95% CI)^a^		*P* value	Intraclass correlation (95% CI)^a^		*P* value
2.4	43	.71 (0.53 to 0.83)	<.001	.16 (−0.15 to 0.44)		.88	.14 (−0.16 to 0.42)		0.92
3.2	43	.80 (0.67 to 0.89)	<.001	.22 (−0.09 to 0.49)		.36	.28 (−0.02 to 0.53)^b^		.04
4.0	42	.98 (0.97 to 0.99)	<.001	.70 (0.50 to 0.83)		<.001	.94 (0.90 to 0.97)		<.001
5.6	41	.99 (0.976 to 0.993)	<.001	.96 (0.93 to 0.98)		<.001	.39 (0.10 to 0.62)^b^		.03
6.4	38	.95 (0.91 to 0.98)	<.001	.93 (0.86 to 0.96)		<.001	.53 (0.26 to 0.72)^b^		.01
7.2	30	.99 (0.97 to 0.992)	<.001	.98 (0.95 to 0.99)		<.001	.66 (0.43 to 0.81)^b^		<.001
Overall	N/A^c^	.97 (0.965 to 0.978)	<.001	.72 (0.65 to 0.77)		<.001	.62 (0.54 to 0.69)^b^		<.001

^a^Three decimal places were used for clarity when necessary.

^b^n=42.

^c^N/A: not applicable.

**Table 3:** Lin’s concordance coefficient.

Walking speed (km/h)	n	Lin’s concordance coefficient (95% CI)^a^		
		PiezoRx	Actigraph	Omron
2.4	43	0.69 (0.51 to 0.81)	0.02 (−0.02 to 0.05)	0.05 (−0.02 to 0.13)
3.2	43	0.80 (0.67 to 0.89)	0.12 (−0.01 to 0.25)	0.21 (0.09 to 0.32)
4.0	42	0.98 (0.96 to 0.99)	0.67 (0.49 to 0.80)	0.92 (0.86 to 0.95)
5.6	41	0.99 (0.97 to 0.991)	0.95 (0.90 to 0.97)	0.26 (0.09 to 0.41)
6.4	38	0.95 (0.91 to 0.97)	0.92 (0.85 to 0.96)	0.53 (0.34 to 0.68)
7.2	30	0.98 (0.96 to 0.99)	0.96 (0.93 to 0.98)	0.66 (0.45 to 0.80)
Overall	N/A^b^	0.97 (0.96 to 0.98)	0.64 (0.59 to 0.68)	0.61 (0.55 to 0.66)

^a^Three decimal places were used for clarity when necessary.

^b^N/A: not applicable.

**Table 4:** Absolute step difference between manually counted steps and steps measured using each device during treadmill walking.

Walking speed (km/h)	n	PiezoRx		Actigraph		Omron	
		Absolute step difference, % (SD)^a^	*P* value	Absolute step difference, % (SD)^a^	*P* value	Absolute step difference, % (SD)^a^	*P* value
2.4	43	6.2 (10.6)	.62	66.6 (22.3)	<.001	61.3 (37.1)	<.001
3.2	43	2.5 (5.4)	.81	18.0 (17.2)	<.001	19.2 (27.7)	.04
4.0	42	1.0 (1.0)	1.00	3.0 (6.5)	.20	1.8 (2.0)	1.00
5.6	41	0.8 (0.6)	1.00	1.3 (1.5)	.98	4.6 (16.3)	.05
6.4	38	1.0 (1.9)	1.00	1.5 (2.4)	.84	2.7 (8.6)	.30
7.2	30	1.1 (1.2)	.86	1.4 (1.4)	.98	2.8 (7.6)	.28
Overall	N/A^b^	2.2 (5.4)	.87	15.9 (26.8)	.50	15.0 (29.0)	.48

^a^Three decimal places were used for clarity when necessary.

^b^N/A: not applicable.

**Table 5:** Reliability of the PiezoRx in measuring step count and moderate-to-vigorous physical activity.

Walking speed (km/h)	n	Step count		Moderate-to-vigorous physical activity		
		Intraclass correlation (95% CI)	*P* value	Spearman correlation coefficient	*P* value	Absolute difference (sec/stage), % (SD)
2.4	25	.62 (.48 to.75)	<.001	.90	<.001	6.1 (17.3)
3.2	23	.59 (0.45 to 0.72)	<.001	.93	<.001	23.8 (55.1)
4.0	25	.33 (0.17 to 0.50)	.01	.92	<.001	22.8 (70.6)
5.6	23	.27 (0.12 to 0.49)	.02	.53	.02	2.4 (3.7)
6.4	23	.993 (0.988 to.996)	<.001	.78	.002	1.1 (2.4)
7.2	20	.98 (0.97 to 0.99)	<.001	.58	.01	3.0 (3.8)
Overall	N/A	.88 (0.86 to 0.90)	<.001	.95	<.001	10.4 (39.3)

**Table 6:** Sensitivity and specificity rate of each activity monitor in measuring moderate-to-vigorous physical activity.

Monitor	Sensitivity (%)	Specificity (%)
PiezoRx (100/120)	70.9	91.7
PiezoRx (110/130)	90.3	79.2
PiezoRx (height adjusted)	82.1	94.3
PiezoRx (height and fitness adjusted)	81.6	91.0
ActiGraph	83.0	89.6

**Figure 2:**
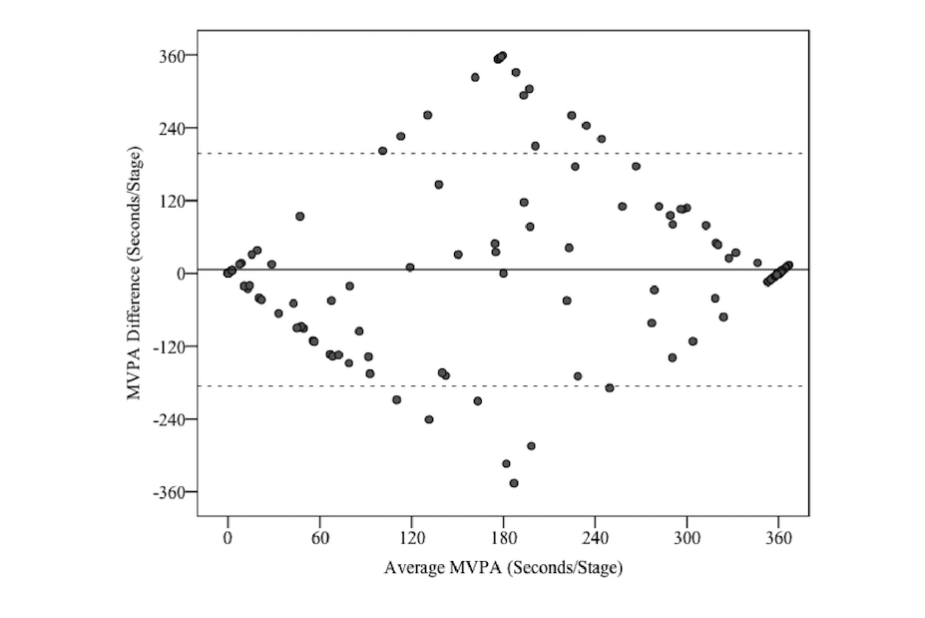
Bland-Altman plot analysis for indirect calorimetry and the height-adjusted PiezoRx-determined moderate-to-vigorous-intensity physical activity (MVPA).

The PiezoRx (110/130) had the highest sensitivity percentage, whereas the height-adjusted PiezoRx had the highest specificity percentage (see Table 6). As shown in Figure 2, BAPs revealed a mean bias of 6.0 seconds (95% LoA: −186, 198) for the height-adjusted PiezoRx in measuring MVPA relative to the metabolic cart.

In the sample of individuals in which the height-adjusted and height+fitness-adjusted PiezoRxs were set at the same step rate thresholds (n=25), SCC of the devices in measuring MVPA was 0.95 (*P*<.001), as shown in Table 5. The interinstrument reliability was highest during the slowest stages (2.4-4.0 km/h; SCC>.90). However, the absolute MVPA differences were lowest during the faster stages (5.6-7.2 km/h; ≤3.0 sec per stage).

## Discussion

The objective of this study was to assess the validity and interinstrument reliability of a medical-grade PA monitor in measuring both step count and MVPA in a laboratory setting. Compared with manual step counting, the PiezoRx had the strongest correlations and a lower percent error than commonly used research grade monitors. When the PiezoRxs MVPA step rate thresholds were adjusted for height, the performance of the device in measuring intensity-related PA was similar to that of a research grade accelerometer.

The previous laboratory-based PiezoRx step count validation studies have shown that the device was accurate when used in a variety of populations [14-16]. Specifically, the 4-stage (1.4-14.1 km/h) step count evaluation of the SC-StepMX (previous version of the PiezoRx) by Colley et al [15] showed an excellent agreement with the manual step counting (r^2^=0.97; APE=−0.2%). The findings of this study were in accordance with such findings for step count (APE=2.2%). Moreover, the PiezoRxs was found as a reliable measurement tool for step count across a variety of walking speeds (overall ICC=.88). ICCs were higher in the earlier (1 and 2) and later (5 and 6) stages but were lowest (ICC<.50) during stages 3 and 4 (4.0 and 5.6 km/h). However, the middle stages had the lowest APE (~1.0%) when the counted steps obtained using the PiezoRx were compared with the manually counted steps, and such result indicates that the low ICC may be due to the similarity in the number of steps in these stages (ie, less variance) with an average of 120±7 and 128±7 spm, respectively. Overall, the device is a reliable measurement tool for step count, particularly at slower walking cadence in which the PA monitoring devices are more likely to have difficulty measuring.

The only laboratory-based investigation on the validity of the PiezoRx device in measuring MVPA was conducted in children and young adults [14]. However, the PiezoRx has previously been used to measure MVPA in older adults [27], and it will be used in an upcoming randomized control trial aimed at increasing the number of steps per day of hospital employees [28]. Saunders et al [14] have shown that the device is a valid measurement tool for MVPA (r^2^=0.64), and it was even more accurate when the step rate thresholds were increased from 100/120 to 110/130 (r^2^=0.82), and this was identical to the correlation between indirect calorimetry and the ActiGraph. Such results are consistent with those of this study in which the height-adjusted PiezoRx and the ActiGraph were similarly correlated to indirect calorimetry (SCC=.80). BAP comparing MVPA measured via indirect calorimetry and the PiezoRx (height adjusted) resembles a diamond shape with a large cluster of data points with a mean difference of 0 (see Figure 2). The diamond shape is due to the dichotomous nature of measuring MVPA through walking stages in which the PiezoRx may incorrectly identify a stage as 360 sec of MVPA, whereas the metabolic cart did not detect any MVPA (average MVPA of 180 sec). In relation to this, the capability of the height-adjusted PiezoRx and ActiGraph in distinguishing each walking stage as MVPA or not was similarly good, as shown by their sensitivity rates (PiezoRx, H: 82%; ActiGraph: 83%) and specificity values (PiezoRx, H: 94%; ActiGraph: 90%). However, the PiezoRx devices set at 100/120, 110/130, and height+fitness adjusted were not significantly different (SCC: 0.74-0.80) than the height-adjusted PiezoRx.

The Yamax Digiwalker has been used as a criterion for comparing the capability of other devices in counting steps in free-living environments [29,30]. A previous research in adults has shown that the error rate of the Yamax Digiwalker (20.5%) was remarkably higher than that of the SC-StepMX (APE=0.2%) [15]. In addition, the Omron HJ-720 is more accurate than the ActiGraph GT3X+ and the Polar Active Accelerometer in laboratory-based and free-living conditions [29]. This study showed that the Omron and ActiGraph GT3X had similar validity in measuring step count (~15% APE).

As previously mentioned, the PiezoRx had a similar or higher accuracy rate than accelerometry in measuring intensity-related PA. Accelerometers may be costly and require proprietary software programs to access specific device data. Although accelerometers may be convenient for researchers, their usability among the general population is limited. After an explanation of the device’s features and a 2-week monitoring, the performance of the PiezoRx was good among a diverse sample of adults and older adults [13]. More recently, the PiezoRxD has been developed which uses Bluetooth technology for uploading individual device data through a patient-focused mobile phone app that serves as a platform for health care providers and exercise professionals to access and provide their respective behavior changes or behavior maintenance approaches. In relation to this, the technology behind the determination of step count and MVPA is identical between the PiezoRx and PiezoRxD. Accurate devices that permit real-time step and MVPA monitoring may assist the general public in increasing their activity levels and may allow health care professionals and exercise professionals to objectively monitor and prescribe PA to their patients or clients with a goal of having more Canadians adhere to the PA guidelines [31].

The use of a submaximal assessment of aerobic fitness may be considered a limitation of this study. However, the single-stage treadmill protocol has been previously validated [21], and its use was to classify the aerobic fitness of the participants according to different VO_2 max_ ranges (excellent, very good, good, fair, or poor), which would be conducted in a clinical setting. As such, we believe that this limitation is minor relative to the validation of this device for clinical use. One limitation of PA studies is that they primarily appeal to physically active populations, potentially biasing the testing cohort to have a higher fitness compared with the general public. The population of this study had a wide range of BMI (17.9-42.5 kg/m^2^) and predicted VO_2 max_ (20.4-58.9 mL/kg/min); therefore, our results are generalizable to the general public. Although the sample of the study was heterogeneous in nature and was designed to be representative of a typical patient population (56% answered “yes” to at least one question on the PAR-Q+), the participants were aged between 20 and 64 years; thus, the results may not be extrapolated to young or older adults. Hence, future studies should investigate the validity and reliability of the PiezoRx in measuring step count and MVPA in these populations who typically have shorter stride lengths and higher stride rates at a given walking velocity. In addition, the accuracy of the PiezoRx should be compared across a variety of movements, such as running, incline walking, stair walking, etc, that may affect the accuracy of this device.

The PiezoRx medical-grade PA monitor is a valid and reliable tool for measuring step count and intensity-related PA among a diverse sample of adults in a laboratory setting. The accuracy of the device in measuring MVPA may be similar to that of a research grade monitor, and it may be even more accurate than other frequently used PA monitors in measuring step counts. The PiezoRx may be a cost-effective alternative to research grade monitors that are used by primary care providers and exercise professionals in providing step-based exercise prescriptions.

## Abbreviations

APE: absolute percent error
BAP: Bland-Altman plots
BMI: body mass index
CSEP: Canadian Society for Exercise Physiology
ICC: intraclass correlation
LCC: Lin’s concordance coefficient
LoA: limits of agreement
METs: metabolic equivalents
MPA: moderate-intensity aerobic physical activity
MVPA: moderate-to-vigorous-intensity physical activity
PA: physical activity
PAR-Q+: Physical Activity Readiness Questionnaire Plus
PASB-Q: Physical Activity and Sedentary Behaviour Questionnaire
RM-ANOVA: repeated measures analysis of variance
SCC: Spearman correlation coefficient
VPA: vigorous intensity physical activity

## References

[1] Warburton DER, Nicol CW, Bredin SSD (2006). Health benefits of physical activity: the evidence. CMAJ.

[2] Colley RC, Garriguet D, Janssen I, Craig CL, Clarke J, Tremblay MS (2011). Physical activity of Canadian adults: accelerometer results from the 2007 to 2009 Canadian Health Measures Survey. Health Rep.

[3] Bravata DM, Smith-Spangler C, Sundaram V, Gienger AL, Lin N, Lewis R, Stave CD, Olkin I, Sirard JR (2007). Using pedometers to increase physical activity and improve health: a systematic review. JAMA.

[4] Qiu S, Cai X, Chen X, Yang B, Sun Z (2014). Step counter use in type 2 diabetes: a meta-analysis of randomized controlled trials. BMC Med.

[5] Dasgupta K, Rosenberg E, Joseph L, Cooke AB, Trudeau L, Bacon SL, Chan D, Sherman M, Rabasa-Lhoret R, Daskalopoulou SS, SMARTER Trial Group (2017). Physician step prescription and monitoring to improve ARTERial health (SMARTER): A randomized controlled trial in patients with type 2 diabetes and hypertension. Diabetes Obes Metab.

[6] O'Brien MW, Shields CA, Oh PI, Fowles JR (2017). Health care provider confidence and exercise prescription practices of Exercise is Medicine Canada workshop attendees. Appl Physiol Nutr Metab.

[7] Evenson KR, Goto MM, Furberg RD (2015). Systematic review of the validity and reliability of consumer-wearable activity trackers. Int J Behav Nutr Phys Act.

[8] Abel M, Hannon J, Mullineaux D, Beighle A (2011). Determination of step rate thresholds corresponding to physical activity intensity classifications in adults. J Phys Act Health.

[9] Marshall SJ, Levy SS, Tudor-Locke CE, Kolkhorst FW, Wooten KM, Ji M, Macera CA, Ainsworth BE (2009). Translating physical activity recommendations into a pedometer-based step goal: 3000 steps in 30 minutes. Am J Prev Med.

[10] Beets MW, Agiovlasitis S, Fahs CA, Ranadive SM, Fernhall B (2010). Adjusting step count recommendations for anthropometric variations in leg length. J Sci Med Sport.

[11] Rowe DA, Welk GJ, Heil DP, Mahar MT, Kemble CD, Calabró MA, Camenisch K (2011). Stride rate recommendations for moderate-intensity walking. Med Sci Sports Exerc.

[12] Sawyer BJ, Blessinger JR, Irving BA, Weltman A, Patrie JT, Gaesser GA (2010). Walking and running economy: inverse association with peak oxygen uptake. Med Sci Sports Exerc.

[13] O'Brien MW, Wojcik WR, D'Entremont L, Fowles JR (2018). Validation of the PiezoRx® Step Count and Moderate to Vigorous Physical Activity Times in Free Living Conditions in Adults: A Pilot Study. Int J Exerc Sci.

[14] Saunders TJ, Gray CE, Borghese MM, McFarlane A, Mbonu A, Ferraro ZM, Tremblay MS (2014). Validity of SC-StepRx pedometer-derived moderate and vigorous physical activity during treadmill walking and running in a heterogeneous sample of children and youth. BMC Public Health.

[15] Colley RC, Barnes JD, Leblanc AG, Borghese M, Boyer C, Tremblay MS (2013). Validity of the SC-StepMX pedometer during treadmill walking and running. Appl Physiol Nutr Metab.

[16] Webber SC, Magill SM, Schafer JL, Wilson KCS (2014). GT3X+ accelerometer, Yamax pedometer and SC-StepMX pedometer step count accuracy in community-dwelling older adults. J Aging Phys Act.

[17] Warburton D, Bredin S, Jamnik V, Gledhill N (2011). Validation of the PAR-Q+ and ePARmed-X+. Health Fitness J Can.

[18] Fowles JR, O'Brien MW, Wojcik WR, d'Entremont L, Shields CA (2017). A pilot study: Validity and reliability of the CSEP-PATH PASB-Q and a new leisure time physical activity questionnaire to assess physical activity and sedentary behaviours. Appl Physiol Nutr Metab.

[19] CSEP (2013). Canadian Society for Exercise Physiology?Physical Activity Training for Health (CSEP-PATH). CSEP, Ottawa, Ont.

[20] Heyward V, Gibson A (2014). Advanced Fitness Assesment and Exercise Prescription. Human Kinetics, Champaign, Ill.

[21] Ebbeling CB, Ward A, Puleo EM, Widrick J, Rippe JM (1991). Development of a single-stage submaximal treadmill walking test. Med Sci Sports Exerc.

[22] Freedson PS, Melanson E, Sirard J (1998). Calibration of the Computer Science and Applications, Inc. accelerometer. Med Sci Sports Exerc.

[23] Slaght J, Sénéchal M, Hrubeniuk TJ, Mayo A, Bouchard DR (2017). Walking Cadence to Exercise at Moderate Intensity for Adults: A Systematic Review. J Sports Med (Hindawi Publ Corp).

[24] Tudor-Locke C, Han H, Aguiar EJ, Barreira TV, Schuna JM, Kang M, Rowe DA (2018). How fast is fast enough? Walking cadence (steps/min) as a practical estimate of intensity in adults: a narrative review. Br J Sports Med.

[25] Bland JM, Altman DG (1986). Statistical methods for assessing agreement between two methods of clinical measurement. Lancet.

[26] Lin LI (1989). A concordance correlation coefficient to evaluate reproducibility. Biometrics.

[27] McLellan AG, Slaght J, Craig CM, Mayo A, Sénéchal M, Bouchard DR (2018). Can older adults improve the identification of moderate intensity using walking cadence?. Aging Clin Exp Res.

[28] Mitchell M, White L, Oh P, Kwan M, Gove P, Leahey T, Faulkner G (2016). Examining Incentives to Promote Physical Activity Maintenance Among Hospital Employees Not Achieving 10,000 Daily Steps: A Web-Based Randomized Controlled Trial Protocol. JMIR Res Protoc.

[29] Lee JA, Williams SM, Brown DD, Laurson KR (2015). Concurrent validation of the Actigraph gt3x+, Polar Active accelerometer, Omron HJ-720 and Yamax Digiwalker SW-701 pedometer step counts in lab-based and free-living settings. J Sports Sci.

[30] Tully MA, McBride C, Heron L, Hunter RF (2014). The validation of Fibit Zip™ physical activity monitor as a measure of free-living physical activity. BMC Res Notes.

[31] Tremblay MS, Warburton DER, Janssen I, Paterson DH, Latimer AE, Rhodes RE, Kho ME, Hicks A, Leblanc AG, Zehr L, Murumets K, Duggan M (2011). New Canadian physical activity guidelines. Appl Physiol Nutr Metab.

