# Correction: Medical-Grade Physical Activity Monitoring for Measuring Step Count and Moderate-to-Vigorous Physical Activity: Validity and Reliability Study

**DOI:** 10.2196/12576

**Published:** 2019-01-03

**Authors:** Myles William O'Brien, William Robert Wojcik, Jonathon Richard Fowles

**Affiliations:** ^1^ Centre of Lifestyle Studies, School of Kinesiology Acadia University Wolfville, NS Canada; ^2^ Division of Kinesiology Dalhousie University Halifax, NS Canada

The Authors of  “Medical-Grade Physical Activity Monitoring for Measuring Step Count and Moderate-to-Vigorous Physical Activity: Validity and Reliability Study” (JMIR Mhealth Uhealth 2018;6(9):e10706) mistakenly represented the Yamax Digiwalker in the Discussion section. Unlike some of Yamax’s newer devices (ie, Yamax EX-510), the Yamax Digiwalker is a spring-levered pedometer and not a piezoelectric pedometer.

Thus, the following sentence has been removed from the Discussion:

Similar to the PiezoRx, the Yamax also uses a piezoelectric sensor, which is consistent with this study.

The correction will appear in the online version of the paper on the JMIR website on January 3, 2019, together with the publication of this correction notice. Because this was made after submission to PubMed, PubMed Central, and other full-text repositories, the corrected article also has been resubmitted to those repositories.

